# Complete Genome Sequence of Viral Hemorrhagic Septicemia Virus Isolated from an Olive Flounder in South Korea

**DOI:** 10.1128/genomeA.00681-13

**Published:** 2013-09-05

**Authors:** Jong-Oh Kim, Wi-Sik Kim, Toyohiko Nishizawa, Myung-Joo Oh

**Affiliations:** Department of Aqualife Medicine, Chonnam National University, Yeosu, South Koreaa; The Fisheries Science Institute, Chonnam National University, Yeosu, South Koreab

## Abstract

Viral hemorrhagic septicemia virus (VHSV) is a seriously problematic pathogen in olive flounder (*Paralichthys olivaceus*) aquaculture farms in South Korea. Here, we report the complete genome sequence of VHSV which was isolated from spleen and kidney tissues of dead fish at an aquaculture farm in 2005. This genome sequence will be useful for virus diagnostics and in comparative analyses with other virus genotypes.

## GENOME ANNOUNCEMENT

Viral hemorrhagic septicemia virus (VHSV) is a problematic pathogen in olive flounder (*Paralichthys olivaceus*) aquaculture farms in South Korea. VHSV is a *Novirhabdovirus* RNA virus with a negative-sense single-stranded RNA (approximately 11 kb) and belongs to the family *Rhabdoviridae* (1). It has six genes in the viral genome: genes encoding a nucleoprotein (N), a polymerase-associated phosphoprotein (P), a matrix protein (M), a glycoprotein (G), a nonvirion protein (NV), and a large RNA-dependent RNA polymerase (L). Phylogenetic analysis based on the partial G gene demonstrated that the virus has four genotypes among the freshwater and marine isolates. Genotype I includes a wide range of viruses from freshwater and marine species in Europe, genotype II is composed of marine isolates from the Baltic Sea, genotype III includes isolates from the British Isles and Scottish waters, and genotype IV contains isolates from the Pacific Ocean (North American, Japanese, and South Korean isolates) (2–8).

FYeosu05, the VHSV strain isolated from an olive flounder cultured in 2005 in Yeosu, South Korea, was propagated in the fathead minnow (FHM) cell line. The nucleotide sequences of VHSV were amplified by reverse transcription-PCR (RT-PCR). Next, the PCR products were purified with the QIAquick gel extraction kit (Qiagen) and ligated with pGEM-T easy vector system (Promega). All transformants were confirmed by restriction enzyme digestion, and the clones were sequenced with an ABI 3730xl DNA sequencer. All sequences were assembled and analyzed manually.

The entire genome size of VHSV isolate FYeosu05 is 11,168 bp, including all coding regions and intergenic sequences. The genome consists of 6 open reading frames (ORFs) arranged in the order 3′-N–P-M-G-NV-L-5′, which is the same order found in other fish rhabdoviruses. It shows >96% identity with genogroup I (99% with strains JF00Ehil and KJ2008, 97% with strain KRRV9822, and 96% with strain MI03GL) and 86% with all other genogroup strains. Among all the VHSV proteins, the RNA polymerase (L) protein is the highest conserved protein (>96% identity), while nonvirion (NV) protein is the most divergent, showing 72 to 100% identity. The genome sequence of VHSV shows a putative polyadenylation motif (AGAT[T/A]GAAAAAAA), which signals the generation of the poly(A) tail to the 3′ end of mRNA and is followed by the nucleotides GGCAC, which is a putative transcription start signal. The 5′ and 3′ untranslated regions (UTRs) are 54 and 101 nucleotides, respectively. This genome sequence will be useful for virus diagnostics and comparative analyses with other virus genotypes.

### Nucleotide sequence accession number.

The complete genome sequence of the VHSV isolate FYeosu05 has been deposited in GenBank under the accession no. KF477302.
